# Complement as an Endogenous Adjuvant for Dendritic Cell-Mediated Induction of Retrovirus-Specific CTLs

**DOI:** 10.1371/journal.ppat.1000891

**Published:** 2010-04-29

**Authors:** Zoltán Bánki, Wilfried Posch, Asim Ejaz, Verena Oberhauser, Suzanne Willey, Christoph Gassner, Heribert Stoiber, Ulf Dittmer, Manfred P. Dierich, Kim J. Hasenkrug, Doris Wilflingseder

**Affiliations:** 1 Department of Hygiene, Microbiology and Social Medicine, Division of Virology, Innsbruck Medical University, Innsbruck, Tirol, Austria; 2 MRC/UCL Centre for Medical Molecular Virology, Division of Infection and Immunity, University College London, London, United Kingdom; 3 Central Institute for Blood Transfusion & Immunological Department, Innsbruck, Tirol, Austria; 4 Institute of Virology, University of Duisburg-Essen, Essen, Germany; 5 Laboratory of Persistent Viral Diseases, Rocky Mountain Laboratories, Hamilton, Montana, United States of America; SAIC-Frederick, United States of America

## Abstract

Previous studies have demonstrated the involvement of complement (C) in induction of efficient CTL responses against different viral infections, but the exact role of complement in this process has not been determined. We now show that C opsonization of retroviral particles enhances the ability of dendritic cells (DCs) to induce CTL responses both *in vitro* and *in vivo*. DCs exposed to C-opsonized HIV *in vitro* were able to stimulate CTLs to elicit antiviral activity significantly better than non-opsonized HIV. Furthermore, experiments using the Friend virus (FV) mouse model illustrated that the enhancing role of complement on DC-mediated CTL induction also occurred *in vivo*. Our results indicate that complement serves as natural adjuvant for DC-induced expansion and differentiation of specific CTLs against retroviruses.

## Author Summary

Upon entering the body, HIV initiates immediate responses of the immune system. The complement system constitutes a first line of defense against HIV and bridges innate and adaptive immunity. Thus, in the acute phase of infection, HIV is coated with complement fragments. Following seroconversion, when HIV-specific antibodies appear, antibodies and complement are deposited onto HIV. Dendritic cells (DCs), the most potent antigen-presenting cells, interact with complement- and Ig-opsonized HIV due to expression of the appropriate receptors. Recently, we found differences with respect to infection of DCs with complement- and Ig-opsonized HIV. We have now investigated whether these differently opsonized HIV preparations also modulated the antigen-presenting capacity of DCs. Since it is extremely difficult to investigate the role of HIV-complement interactions on the induction of virus-specific cytotoxic T lymphocytes (CTLs) *in vivo*, we used the well-characterized Friend virus (FV) mouse model for our *in vivo* studies. We now show that complement opsonization of retroviral particles enhanced the ability of DCs to induce CTL responses against HIV or FV. Thus, our results indicate that complement serves as natural adjuvant for DC-induced expansion and differentiation of specific CTLs against retroviruses.

## Introduction

During the acute phase of HIV-1 infection the immune system responds with a massive, oligoclonal expansion of CD8^+^ T cells [1]. The appearance of virus-specific CTLs correlates with declining viremia during this acute phase of infection, but CTLs are not associated with control of the virus during the chronic phase [2], [3]. Ongoing HIV infection induces a sustained inflammatory response and causes progressive functional defects in CTL populations [4]. A gradual failure of the immune response occurs due to a dramatic loss of CD4^+^ T cells, spontaneous apoptosis of non-infected, activated CD4^+^ and CD8^+^ T cells, induction of T_regs_, escape of virus-specific CD8^+^ T cell recognition by HIV, and destruction of the follicular dendritic cell network [5]. In long-term non-progressors HIV-specific CTLs are suggested to be important mediators of protection due to increased anti-HIV CTL precursor numbers and lower viral burden [6].

Increasing evidence suggests an important role for the complement system in protection against viral infections. For example, C activation contributes not only directly to host protection against viruses by C-mediated lysis or opsonization, but is also essential in priming humoral responses as demonstrated for different viral infections [7]–[9]. More recently, the involvement of the complement system in priming antiviral T cell immunity was highlighted [10]–[12]. Upon infection of C3-deficient mice with influenza virus, a significant impairment in priming of CD4^+^ helper cells and virus-specific cytotoxic T lymphocytes was observed, which resulted in delayed clearance of the infection and increased viral titers [10]. Similarly, the induction and expansion of CD8^+^ T cells during infection with lymphocytic choriomeningitis virus (LCMV) depended on C3 [11]. A further study investigating West Nile virus (WNV) infection in mice deficient for different complement components revealed that the activation of both classical and alternative pathways was required to induce an efficient T cell response [12]. In line with these observations, C3 together with natural antibodies could act as an endogenous adjuvant for vaccine-induced T cell responses [13].

In HIV-1 infections, virions activate the complement system, and are already coated with C fragments at the initial stages of infection [14], [15]. We recently demonstrated that compared to non-opsonized virus, C-coating of HIV-1 significantly enhanced the infection of DCs *in vitro* through complement receptor type 3 (CR3, CD11b/CD18) and CR4 (CD11c/CD18), which also resulted in a different internalization pattern [14], [16]. Thus, C-opsonization of retroviruses could have profound consequences on the antigen-presenting capacity of DCs and the subsequent immune response.

Since it is extremely difficult to investigate the role of HIV-complement interactions on the induction of virus-specific CTLs *in vivo* we used the well-characterized Friend virus (FV) mouse model for *in vivo* studies. FV is a retroviral complex consisting of two viruses: a non-pathogenic replication-competent helper virus called Friend murine leukemia virus (F-MuLV) and a pathogenic replication-defective spleen focus-forming virus (SFFV) [17]. Infection of adult mice with this complex results in polyclonal proliferation of erythroid precursor cells causing massive splenomegaly. Disease progresses to lethal erythroleukemia in susceptible mouse strains, whereas resistant mouse strains are able to control, but never completely eradicate infection. A chronic infection develops, which is associated with the induction of T_regs_ that suppress effector functions of virus-specific CTLs [18], [19].

Here, we found that DCs exposed to C-opsonized HIV induced a more pronounced and functional virus-specific CD8^+^ T cell response *in vitro* compared to the priming with DCs exposed to non-opsonized HIV. This DC-mediated, C-dependent priming of virus-specific CTLs was confirmed *in vivo* using the FV model. Our *in vitro* and *in vivo* observations provide the first evidence that DCs along with complement opsonization account for effective CTL induction upon viral infections.

## Results

### Repeated prime-boosting with HIV-C-exposed DCs triggers CD8^+^ T cell proliferation

**Figure 1:**
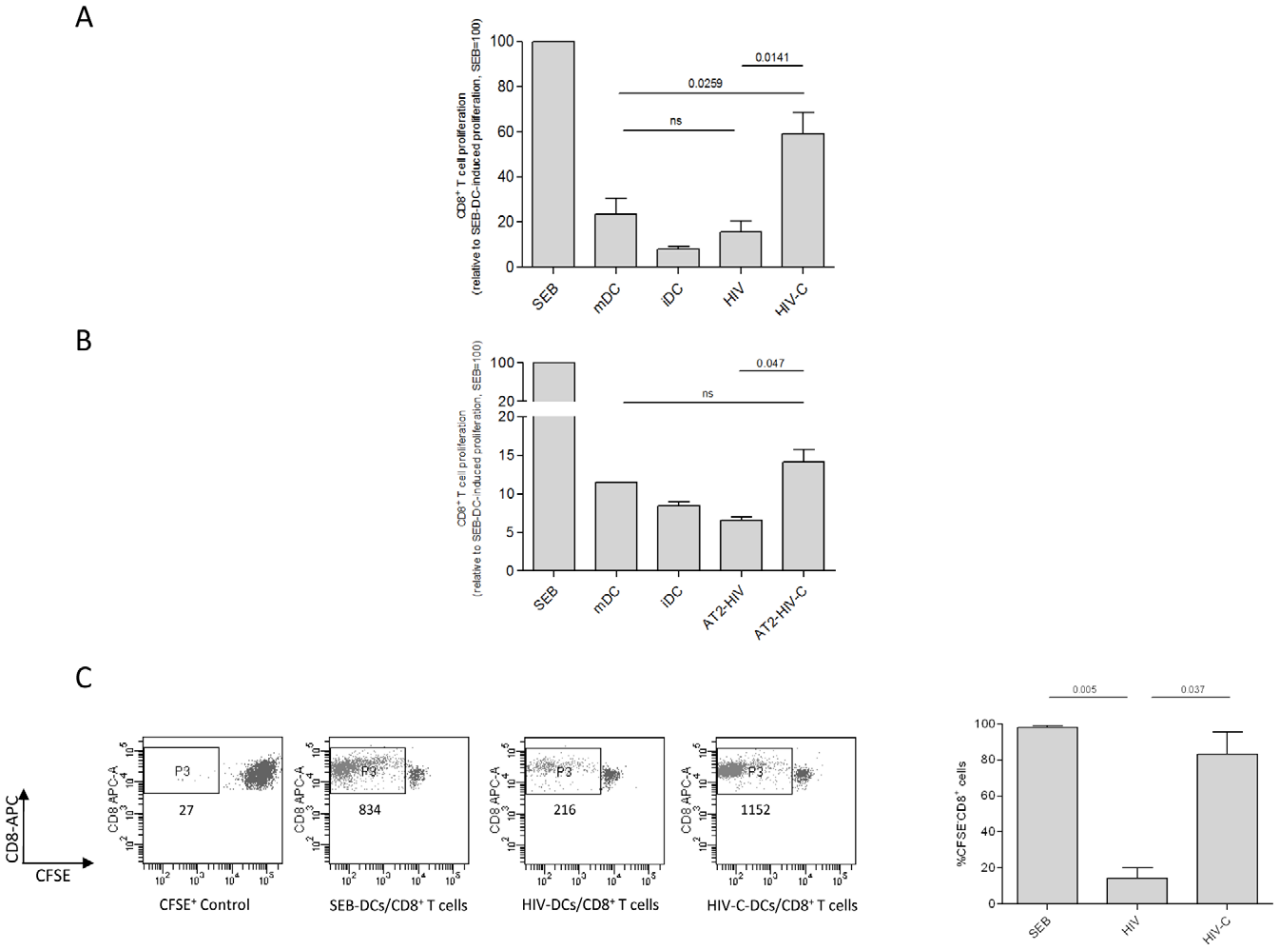
Proliferation of HIV-C-DC-primed CD8^+^ T cells. Complement-opsonization of HIV (**A**) or AT2-inactivated HIV (**B**) and subsequent loading of DCs initiates proliferation of CD8^+^ T cells as measured by counting CD8^+^ T cells over a one minute period of time (**A**) and by CFSE-staining of CD8^+^ T cells (**C**). As positive control, DCs were exposed to the superantigen SEB, which induced strong proliferation of CD8^+^ T cells. The response to SEB was set at 100%. (**A**) No or low proliferation comparable to iDC-primed CD8^+^ T cells was mediated by DCs exposed to non-opsonized HIV (HIV). Cytokine-stimulated mDC-primed CD8^+^ T cells showed low proliferation, while HIV-C-exposed DCs induced about 60% proliferation compared to SEB-DC-primed CD8^+^ T cells. Columns represent mean values of CD8^+^ T cells from 5 donors stimulated with either BaL or 92UG037. Data were analyzed by unpaired Student's t-test. (**B**) Prime-boosting by AT2-HIV-C-loaded DCs caused proliferation of autologous CD8^+^ T cells, albeit weaker than with HIV-C-exposed DCs. In contrast, DCs loaded with AT2-HIV did not induce proliferation of CD8^+^ T cells at all. This was repeated four times with HIV strains 92BR030 or 92UG037. (**C**) Complement-mediated proliferation of CD8^+^ T cells was confirmed with 5 donors by staining cells with 2.5 µM CFSE prior addition of loaded DCs (n = 5). Left panel: dot plot of CD8^+^ T cells exposed three times to SEB-, HIV- or HIV-C-loaded DCs, and a CFSE^+^ control. The amount of cells in the live CD8^high^ population (P3) is shown in the dot plot for one representative donor. Right panel: mean percentages of CFSE^−^ [proliferated]/CD8^+^ T cells of SEB-, HIV- or HIV-C-DC-primed CD8^+^ T cells from 5 donors. Data were analyzed by unpaired Student's t-test.

Naive CD8^+^ T cells were primed-boosted three times with loaded DCs to determine if complement opsonization of HIV exerted an influence on the antigen-presenting capacity of DCs. To mimic the *in vivo* situation, where HIV is opsonized with complement fragments at the beginning of infection, we opsonized live virus with complement (HIV-C) prior to incubation with cytokine-stimulated monocyte-derived DCs. The effect of HIV-C on DCs was compared to DCs exposed to non-opsonized HIV (HIV) or HIV opsonized with complement-inactivated serum (HIV-hiC) to confirm the complement-mediated effects and exclude other factors possibly present in serum such as natural Abs. The opsonization pattern of the single HIV-preparations is shown in Supplementary Figure S1A. Furthermore, DCs stimulated with either superantigen Staphylococcal Enterotoxin B (SEB-DCs) or a cytokine-cocktail (IL-1β, IL-6, TNF-α, PGE_2_) (mDCs) and immature DCs (iDCs) were also included in the experiments. Analysis of relative CD8^+^ T cell concentrations showed that DCs loaded with complement-opsonized HIV triggered significantly more proliferation than those loaded with non-opsonized HIV (p = 0.0141) (Fig. 1A) and HIV-Ig or HIV-hiC (not shown). The CD8^+^ T cell proliferation induced by cytokine-stimulated mDCs, which served as control for the HIV- or HIV-C-mediated effects, was intermediate and significantly lower than that mediated by HIV-C-DCs (p = 0.0259), but not HIV-DCs (not significant, ns) (Fig. 1A). CD8^+^ T cell proliferation induced by cytokine-stimulated mDCs and iDCs were included as negative controls (Fig. 1A). Fig. 1A summarizes the relative mean expansion of CD8^+^ T cells from 5 donors and two different virus strains (BaL, 92UG037). Since using live virus and cultivating DCs after exposure to HIV in co-culture with the CD8^+^ T cells, we excluded that the HIV-C-DC-mediated expansion of CD8^+^ T cells was exclusively due to a better productive infection of the DCs compared to HIV-DCs. We previously reported that C-opsonization of HIV enhances DC infection at least 2 to 3-fold compared to non-opsonized HIV [14], [16]. Thus, the prime-boost experiment was additionally performed using same DC loading concentrations (25 ng p24/ml) as live virus of non-/hiC- or C-opsonized AT2-inactivated R5- (92UG037) and R5X4 (93BR020)-tropic HIV-1 preparations. Following three prime-boost cycles, DCs loaded with AT2-inactivated HIV-C induced significantly greater CD8^+^ T cell proliferation than DCs loaded with AT2-inactivated, non-opsonized HIV (Fig. 1B). However, the expansion of CD8^+^ T cells mediated by DCs loaded with AT-2-inactivated HIV-C was lower than that induced by DCs loaded with live HIV-C (compare Fig. 1A and 1B).

CD8^+^ T cell proliferation was also analyzed by labeling cells with CFSE prior to the first priming with loaded DCs. Flow cytometric analyses confirmed that stimulation by DCs loaded with complement-opsonized HIV induced significantly greater proliferation of CD8^+^ T cells compared to non-opsonized HIV or HIV-hiC (Figure S1B). Representative dot plots (Fig. 1C, left panel) and mean data (Fig. 1C, right panel) showed that HIV-C-DCs mediated significantly increased T cell expansion that was at least 4-fold higher compared to HIV-DCs, which induced only a low proliferation rate.

### IFN-γ secretion is increased in CD8^+^ T cell cultures after repeated stimulation with C-HIV loaded DCs

**Figure 2:**
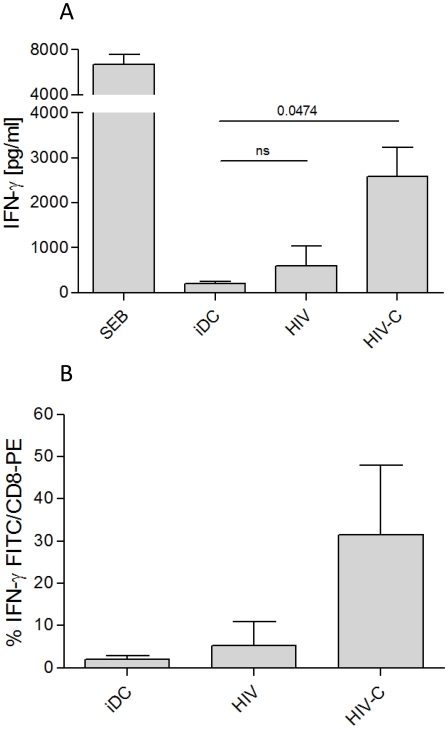
IFN-γ secretion of HIV-C-DC-primed CD8^+^ T cells. IFN-γ secretion by CD8^+^ T cells was measured by OPTEia (**A**) or by an IFN-γ secretion assay (**B**) following the second boost with DCs exposed to LPS or differentially opsonized HIV-1 preparations as indicated. iDC-primed CD8^+^ T cells were used as negative control for IFN-γ secretion. HIV-C-DCs but not HIV-DCs also induced IFN-γ secretion by CD8^+^ T cells following the third boost (not shown). SEB-DCs induced high IFN-γ secretion of CD8^+^ T cells (**A**). In **A**, bars represent means (+SD) from four different CD8^+^ T cell donors stimulated with DCs exposed to differentially opsonized R5- or X4-tropic HIV (92BR030, BaL, NL4-3). In **B**, bars represent mean percentages of IFN-γ-secreting CD8^+^ T cells after re-stimulation with iDCs or DCs exposed to HIV or HIV-C (BaL, 92BR030). The IFN-γ-secretion assay was performed with T cells from 5 different donors and data were analyzed by unpaired Student's t-test.

Since IFN-γ is a cytokine predominantly secreted by activated CD8^+^ and CD4^+^ effector T cells, we next tested whether the expanded CD8^+^ T cells secreted IFN-γ after repeated priming with loaded DCs. HIV-C-loaded DCs induced significantly higher IFN-γ secretion by CD8^+^ T cell cultures compared to HIV-loaded DCs, which produced no significant IFN-γ above control iDCs (Fig. 2A). The results obtained by ELISA were confirmed by an IFN-γ secretion assay, which showed that HIV-C-DC-boosted CD8^+^ T cells secreted increased amounts of IFN-γ compared to HIV-DC-boosted CD8^+^ T cells (Fig. 2B).

### HIV-C-DC-primed CD8^+^ T cells are antigen-specific

**Figure 3:**
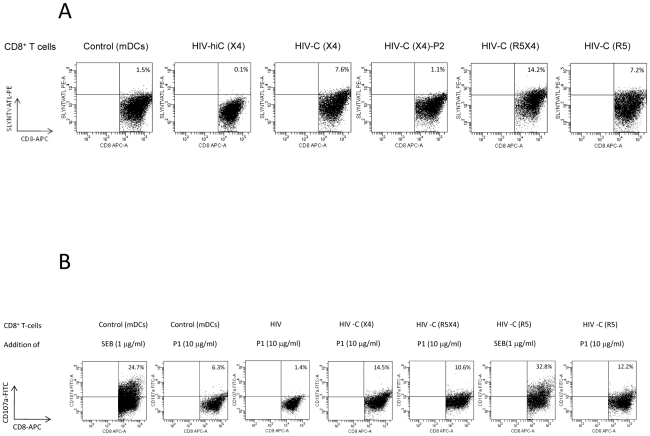
Specificity and degranulation of HIV-C-DC-primed CTLs. (**A**) **Analysis of the specificity of the in vitro generated CTLs by tetramer analyses.** Dead cells were excluded from analyses by 7-AAD staining and FACS plots were gated on CD8^high^ T cell populations. The panel of dot plots shows the specificity by SLYNTVATL-PE tetramer staining (HLA-A02). In **A**, the top line (CD8^+^ T cells) refers to what the cells were primed with. For negative controls CD8^+^ T cells were primed with cytokine-stimulated mDCs and re-stimulated with the HIV(HLA-A02)-specific peptide P1 (Control (mDCs)). As expected, these controls showed only low reactivity with the tetramer (1.5%). Also HIV-hiC-DC-primed CD8^+^ T cells did not recognize the tetramer (HIV-hiC(X4), 0.1%). The percentages of tetramer-positive CD8^+^ T cells cells are outlined in the Figure and only HIV-C-DC-primed CD8^+^ T cells re-stimulated with P1 stained tetramer positive independently of the tropism of the virus used for DC stimulation (HIV-C(X4), HIV-C(R5X4), HIV-C(R5)). 1.1% of HIV-C-DC-CD8^+^ T cells re-stimulated with the control peptide P2 (HIV-C(X4)-P2) were SLYNTVATL-positive comparable to mDC-primed CD8^+^ T cells (HIV-C(X4)-P2). This figure shows a representative tetramer analysis from one donor and three different virus strains (NL4-3, 93BR020, 92BR030). Tetramer analysis was performed 4 times. (**B**) **Degranulation of HIV-C-DC-primed CTLs.** Degranulation was measured by detection of cell surface CD107a after four hours of stimulation with specific peptides as indicated. Cells were gated on live, CD8^high^ populations. SEB-stimulated mDC- or HIV-C(R5)-primed CD8^+^ T cells were used as positive control for degranulation. The top line (CD8^+^ T cells) refers to what the cells were primed with and the bottom line (Addition of) to what they were stimulated with (SEB or peptide P1) and the percentages of CD107a-positive cells are outlined in the figure. Beside SEB, only HIV-C-DC-primed CD8^+^ T cells degranulated upon specific stimulation with P1 independently on the tropism of the virus used (HIV-C(X4)/P1, HIV-C(R5X4)/P1, HIV-C(R5)/P1). Degranulation was measured concomitantly with tetramer analysis and repeated 4 times.

Next we used tetramer analysis to determine whether the DC-stimulated CD8^+^ T cells were HIV-specific. CD8^+^ T cells from an HLA-A02^+^ donor were expanded by stimulation with the differentially loaded DCs and then re-stimulated with HLA-A02-restricted HIV-specific (P1) and control (P2) peptides in the presence of CD3/CD28 beads and IL-2 (100U/ml). We gated on live CD8^high^ T cells. Only a very small proportion of HIV-hiC(X4)- (Fig. 3A) or HIV(X4) (not shown)-DC-primed CD8^+^ T cells reacted with the tetramer (0.1%) whereas 7.6% of the HIV-C(X4)-DC-primed CD8^+^ T cells stained positive following stimulation with the HLA-A02-restricted HIV-specific peptide P1 (Fig. 3A, HIV-C(X4)). In contrast, upon stimulation of HIV-C(X4)-DC-primed CD8^+^ T cells with the control HIV peptide P2 only 1.1% of the cells were stained positive for the tetramer (Fig. 3A, HIV-C(X4)-P2). Dual- and R5-tropic HIV-C-exposed DCs induced 14.2% and 7.2% tetramer positive CD8^+^ T cells upon specific stimulation, respectively (Fig. 3A, HIV-C(R5X4), HIV-C(R5)). Again HIV(R5X4 and R5)-DCs mediated only a low amount (1.4%) of tetramer positive CD8^+^ T cells (not shown). In control experiments, CD8^+^ T cells primed/boosted with cytokine-stimulated mDCs and P1 (Fig. 3A, Control (mDCs)) or P2 (not shown) were analyzed for tetramer-positive cells. Only a small percentage of the mDC-stimulated T cells were tetramer positive (1.5%), which was comparable to that also observed after stimulation of HIV(X4)-C-DC-primed CD8^+^ T cells with the control peptide P2.

### HIV-C-DC-primed and expanded CTLs degranulate upon stimulation with HIV-gag peptides

Next, we assessed the ability of the *in vitro* generated and CD3/CD28-expanded CD8^+^ T cells to degranulate in response to Ag-specific stimulation [20]. By measuring CD107a-mobilization to the cell surface, which occurs upon fusion of the cytolytic membrane, degranulation can be detected by flow cytometry. In all experiments SEB was used as a positive control [21], and a negative control was included to control for spontaneous expression of CD107a. HIV-C-DC-primed CD8^+^ T cells showed significantly greater degranulation upon recognition of the HIV-specific P1 peptide than T cells primed with non-opsonized HIV (Fig. 3B). Degranulation was independent of the tropism of the HIV isolates (Fig. 3B).

### HIV-C-DC primed CD8^+^ T cells elicit anti-HIV-activity

To further characterize the functionality of the *in vitro* generated CD8^+^ T cells, expanded T cells were added to autologous, infected CD4^+^ T cells. Supernatants were taken on several days after co-culture for measurement of p24 by ELISA. The amount of p24 measured in the samples served as readout since a reduced infection of the CD4^+^ T cells indicates an anti-viral effect by the generated CD8^+^ cells.

**Figure 4:**
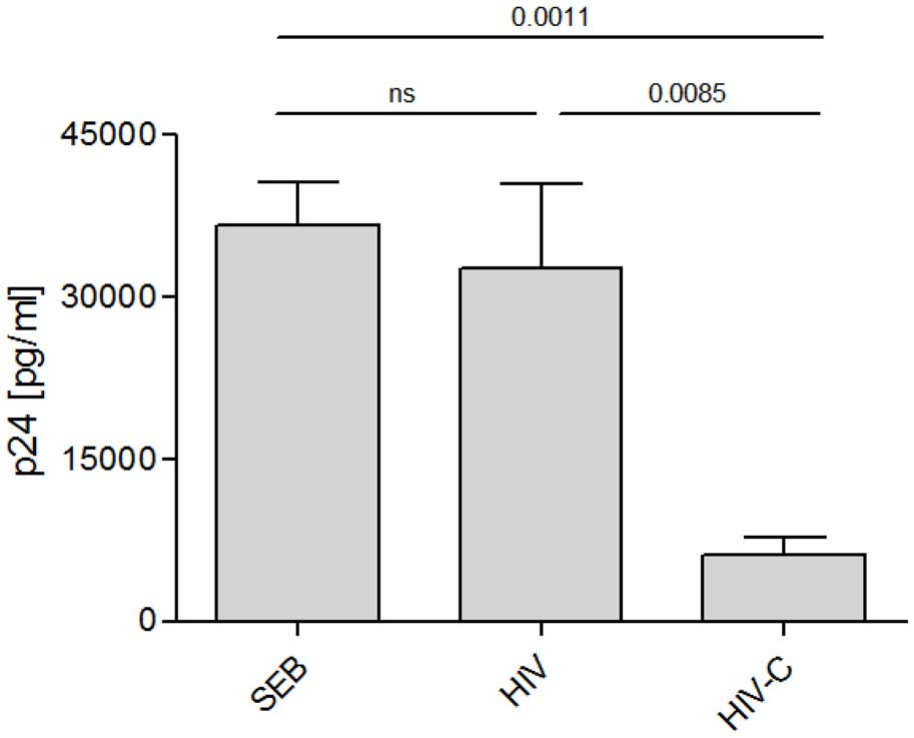
HIV-C-DC-primed CD8^+^ T cells elicit antiviral effects. Inhibition of HIV p24 production by infected autologous CD4^+^ T cells was monitored after incubation with variously stimulated CD8^+^ T cells. Mean p24 values of triplicates (+SD) from one donor on day 11 post addition of HIV-specific CD8^+^ T cells to NL4-3-incubated CD4^+^ T cells are shown. Inhibition by HIV-C-DC-primed CD8^+^ T cells was significantly greater than CD8^+^ T cells primed with non-opsonized HIV (p = 0.0085). Similar results were obtained with cells isolated from 7 different donors using R5-, X4- and R5X4-tropic viral isolates, BaL, 92BR030, 92UG037, NL4-3, 93BR020 (data not shown). A summary of all data in one graph was not feasible due to donor- and virus strain-dependent differences in the infection efficiency, therefore p24 values of one representative donor is shown in this figure.

Indeed, CD8^+^ T cells boosted three times with HIV-C exerted anti-HIV-activity against NL4-3-infected CD4^+^ T cells as the cultures showed a significantly lower p24 value than HIV-infected CD4^+^ T cells (Fig. 4). This result was verified seven times with cells from different donors and different HIV-1 preparations (BaL, 92BR030, 93BR020, data not shown). In addition, the experiments were performed with R5- (BaL) and R5X4- (93BR020)tropic AT-2-inactivated HIV-1 preparations (Figure S2). Also AT2-HIV-C-DC-primed CD8^+^ T cells showed significantly lower p24 values (p = 0.02) than AT2-HIV-DC-stimulated CD8^+^ T cells. Furthermore, in contrast to HIV-DC-CD8^+^ T cells, HIV-C-DC-boosted CD8^+^ T cells also elicited antiviral activity, when stimulated CD4^+^ T cells were infected 3 days prior addition of the specific CTLs, thus inhibiting an already on-going infection of the CD4^+^ T cells. Supplementary Figure S3 shows p24 ELISA values taken from CD4/CD8 T cell supernatants 5 and 9 days post addition of the various CTLs to the already 3 days pre-infected CD4^+^ T cells.

### Complement opsonization triggered activation and proliferation of FV-specific CD8^+^ T cells by virus-loaded bmDCs

To investigate the role of C-opsonization in the induction of CD8^+^ T cell responses *in vivo*, FV infection of mice was chosen as a model. First, we confirmed that opsonization of F-MuLV with complement enhanced infection of DCs as observed with HIV [14], [16]. For this, F-MuLV was incubated *in vitro* in normal mouse serum (NMS) to deposit C3-fragments on the viral surface. Similarly to HIV, C3-deposition was detected by virus capture assay (VCA) (data not shown). Next, we performed infection experiments of mouse bone marrow-derived DCs (bmDCs) with differentially opsonized F-MuLV. Non- and C-opsonized F-MuLV productively infected bmDCs, but significantly higher virus levels were obtained from culture supernatants of DCs infected with F-MuLV-C (data not shown).

**Figure 5:**
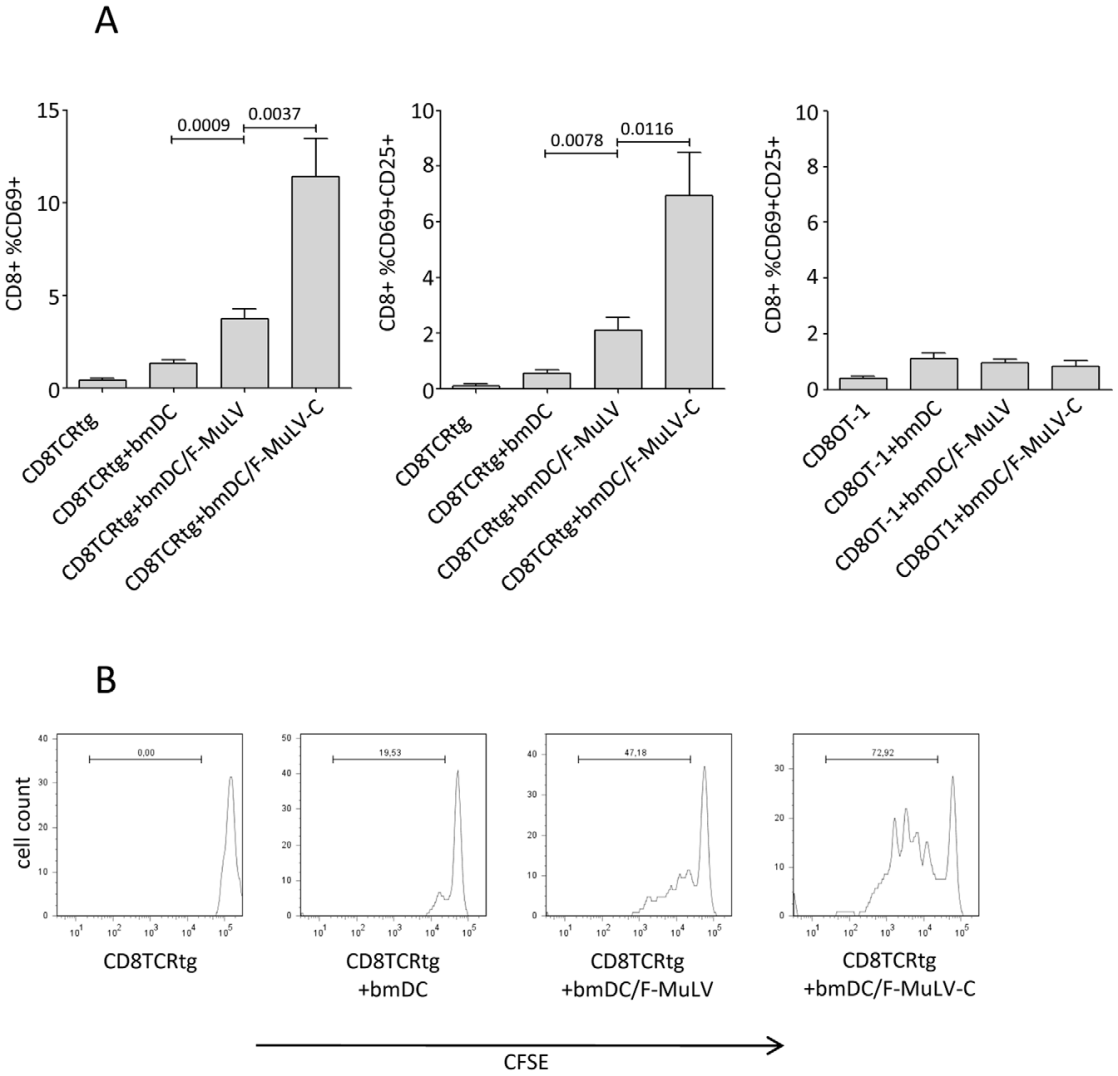
Complement enhances F-MuLV infection of bmDCs and induces activation and proliferation of FV-specific CD8^+^ T cells by virus-loaded DCs. (**A**) Bars indicate mean percentages of CD8^+^ T cells expressing activation markers following stimulation as indicated on the X axis (expression of CD69, left panel; dual expression of CD69 and CD25, middle and right panels). The left two panels show data from virus-specific CD8^+^ T cells while the right panel shows data from non-specific (OT-1) negative control cells. Data were analyzed by unpaired students t-test, n = 10. (**B**) CFSE dilution as measured by flow cytometry was used to analyze proliferation of TCRtg CD8^+^ T cells in response to variously loaded DCs. Data are from one representative of three independent experiments.

We next determined the capacity of bmDCs loaded with non- or C-opsonized F-MuLV to activate transgenic CD8^+^ T cells expressing a T cell receptor specific for an FV gag peptide (TCRtg CD8^+^ T cells) *in vitro* [34]. Co-culture of 1×10^6^ TCRtg CD8^+^ T cells with 1×10^6^ bmDCs exposed to 1000 FFU F-MuLV significantly induced expression of the early activation marker CD69 on CD8^+^ T cells, when compared to control DCs (Fig. 5A). CD8^+^ T cell activation was significantly enhanced when FV-specific CD8^+^ T cells were co-cultured with bmDCs loaded to C-opsonized F-MuLV (Fig. 5A, left). In addition to CD69, CD25 on F-MuLV-C/bmDC-activated CD8^+^ T cells was also expressed (Fig. 5A, middle). Control OVA-specific OT-1 CD8^+^ T cells were not activated by either F-MuLV- or F-MuLV-C-loaded bmDCs indicating that antigen specificity was required for stimulation (Fig. 5A, right).

To study proliferation of FV-specific CD8^+^ T cells induced by virus loaded bmDCs, isolated FV-specific TCRtg CD8^+^ T cells were stained with CFSE prior to co-culture with bmDCs. We found that after 4 days of co-culture both non- and C-opsonized F-MuLV-loaded bmDCs induced proliferation of FV-specific CD8^+^ T cells. However, the proliferation of CD8^+^ T cells induced by F-MuLV-C loaded bmDCs was more pronounced (Fig. 5B).

### Spleen DCs (sDCs) isolated from FV-infected C3-deficient mice show a reduced capacity to induce FV-specific CTLs

**Figure 6:**
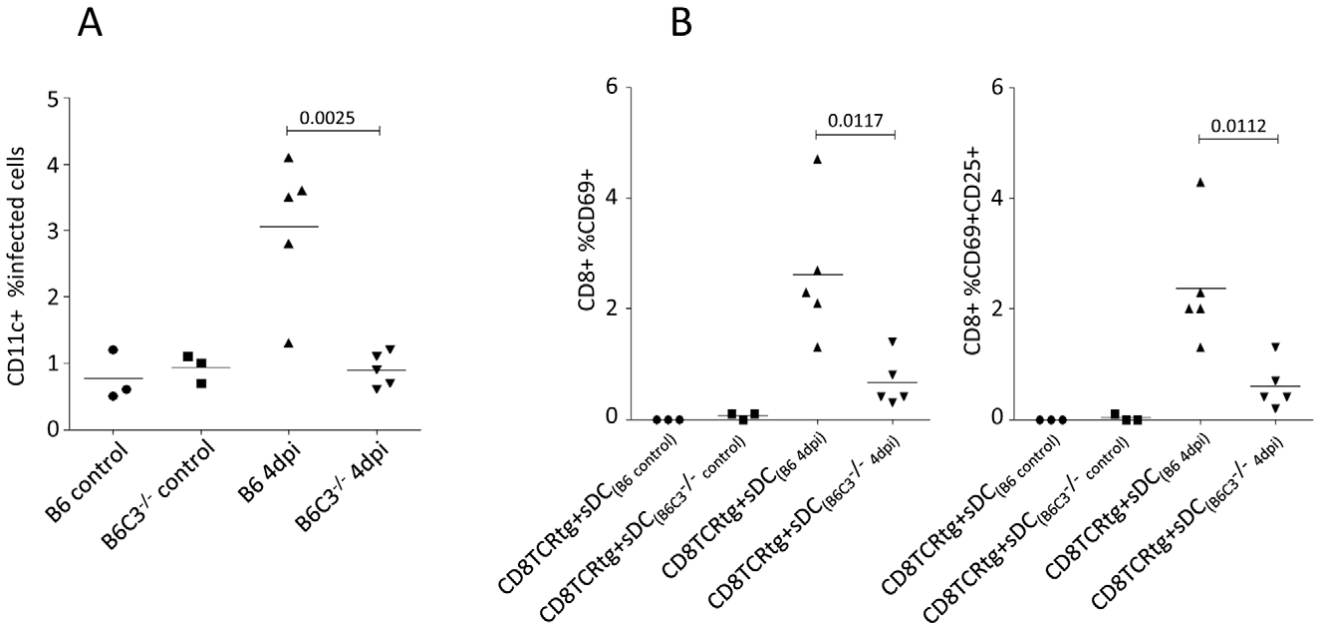
Spleen DCs from FV-infected C3-deficient mice show an impaired capacity to induce FV-specific CTL. (**A**) Infection of CD11c^+^ spleen DCs were analyzed by flow-cytometry from non- and FV-infected B6 wt and C3^−/−^ mice at 4 dpi by expression of viral glycosylated gag protein using mAb clone 34. (**B**) Isolated sDC from non-infected controls (B6 control and B6 C3^−/−^ control) and FV-infected (B6 4 dpi and B6 C3^−/−^ 4 dpi) animals were co-cultured with FV-specific TCRtg CD8^+^ T cells for 24 hours and the activation of FV-specific CTLs was determined by measuring cell-surface expression of CD69 and CD25 on TCRtg CD8^+^ T cells (expression of CD69, left panel; dual expression of CD69 and CD25, right panel). Data were analyzed by unpaired Student's t-test.

To determine whether complement played a role in DC-mediated antigen presentation to CD8^+^ T cells *in vivo*, we investigated B6 wt mice and complement component 3-deficient mice (B6 C3^−/−^) infected with FV. As expected from previous results [22], a significant proportion of splenic CD11c^+^ DCs (sDCs) were infected at 4 days post infection (dpi) (Fig. 6A) as determined by flow-cytometry using mAb clone 34, which specifically stains F-MuLV glycosylated Gag protein expressed on the surface of infected cells. In contrast, there was no significant infection of sDCs from B6 C3^−/−^ mice. Next, we isolated CD11c^+^ sDCs from FV-infected B6 and B6 C3^−/−^ mice after 4 dpi allowing us to investigate their ability to stimulate FV-specific TCRtg CD8^+^ T cells *in vitro*. Compared to DCs from infected wt mice, DCs from infected C3^−/−^ mice had a significantly decreased capacity to activate virus-specific CTLs as determined by the expression of the activation markers CD69 and CD25 on the CD8^+^ T cells (Fig. 6B; expression of CD69, left panel; dual expression of CD69 and CD25, right panel). Thus C3 was important for the infection of DCs *in vivo*, and for their ability to present antigen to CD8^+^ T cells.

### Pronounced FV-infection in C3-deficient mice

**Figure 7:**
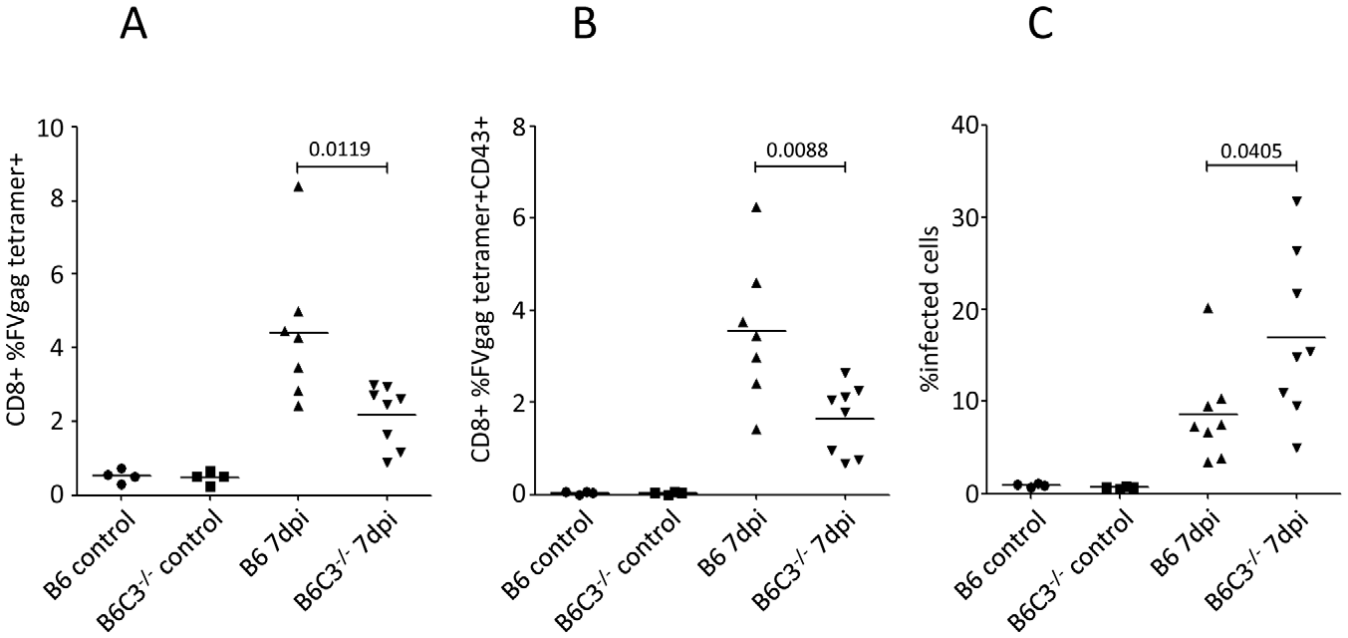
Pronounced FV-infection in C3-deficient mice correlates with a lower frequency of FV-specific CTLs. B6 wt and B6 C3^−/−^ mice were infected with 10000 SFFU of theFV-complex. (**A**) Splenic CD8^+^ T cells were analyzed for FV specificity using gag-specific tetramers. (**B**) Tetramer positive CD8^+^ T cells were analyzed for expression of the effector cell marker CD43. (**C**) FV-infection of spleen cells was monitored by FACS analysis using mAb clone 34. Data were analyzed by unpaired Student's t-test.

To determine if the activation and proliferation of virus-specific CD8^+^ T cells was impaired in C3-deficient mice, splenic CD8^+^ T cells were stained with tetramers. We observed a significantly reduced proportion of tetramer positive CD8^+^ T cells in the C3-deficient mice compared to wt (Fig. 7A). Furthermore, there was significantly reduced expression of the activation-induced isoform of CD43 on CD8^+^ T cells (Fig. 7B). Since previous results indicated that CD8^+^ T cells are critical for recovery from FV infection [23], it was expected that impaired CD8^+^ T cell responses in C3-deficient mice would exacerbate FV infection. Indeed, there were significantly higher proportions of infected spleen cells in B6 C3^−/−^ mice at 7 dpi compared to B6 wt animals (Fig. 7C). These data indicated the importance of C3 for the control of retroviral infection *in vivo*, which correlates with the activation and proliferation of CD8^+^ T cells.

## Discussion

Previous studies have demonstrated the involvement of complement in induction of an efficient CTL response against different viral infections [10]–[12], but the mechanism by which complement affected CTL responses was not elucidated. Our results illustrate an important role for complement in facilitating antigen presentation by DC's to stimulate the activation, expansion, and differentiation of retrovirus-specific CD8^+^ T cells *in vitro* and *in vivo*. The results indicate that complement serves as endogenous adjuvant for DC-induced CTL responses against retroviruses. HIV-specific CTLs generated *in vitro* by repeated prime-boosting with HIV-C-exposed DCs were able to secrete IFN-γ, de-granulated upon stimulation with an HIV-specific HIV-peptide and CD3/CD28 engagement [24] and elicited antiviral activity against HIV-infected autologous CD4^+^ T cells. These CTL priming properties were induced to a much lesser extent by DCs loaded with non-opsonized HIV. Infection of DCs was not the sole pre-requisite for the generation of specific CTLs, since AT-2-inactivated HIV-C-DCs induced a CD8^+^ T cell proliferation, too, and these were also able to elicit antiviral activity against HIV-infected CD4^+^ T cell targets. Experiments using the FV model also revealed antigen-specificity and the enhancing role of complement for DC-mediated CTL induction both *in vitro* and *in vivo*.

The unique role of DCs in priming naïve CD8^+^ T cells in response to exogenous cell-associated as well as endogenously synthesized Ags has been demonstrated [25], [26]. With regard to endogenously synthesized antigens, DCs infected with LCMV elicited strong CTL responses, while LCMV infected macrophages and B cells did not [26]. In the FV model, it has been shown that depletion of CD11c^+^ DCs significantly decreases the frequency of virus-specific CTLs in FV-infected mice [27]. Infection of DCs and the presentation of endogenously synthesized viral antigens processed through the MHC class I pathway might be important during acute viral infection to prime naive CD8^+^ T cells. In line with these observations HIV-C incubation of DCs resulted in enhancement of direct infection of the APCs compared to non-opsonized HIV [14]. Thereby HIV-C-infected APCs could process the endogenous Ags directly through the classic MHC class I processing pathway and thus prime naïve CD8^+^ T cells and induce a virus-specific CTL response. Similarly to HIV, complement-opsonized FV targeted mouse CD11c-positive dendritic cells more efficiently *in vitro* and *in vivo*, which resulted in a more pronounced induction of virus-specific CTLs. Our data strongly support a role for C-opsonization of retroviruses to enhance infection of DCs and therefore facilitate the generation of virus-specific CTLs. This hypothesis is further supported by the reduced FV infection of DCs in C3-deficient mice and the reduced induction of FV-specific CD8^+^ T cells by DCs derived from FV-infected C3-deficient mice. The involvement of neutralizing Abs produced by B cells has been shown to be important in the control of FV infections [17], [28]. Despite C3-deficiences have been demonstrated to have an impact on the maintenance of efficient B cell response as early as 7 dpi, a dominant role an impaired neutralizing B cell response in the elevated FV infection observed in C3-deficient mice is rather unlikely.

Besides presentation of endogenous antigen a more efficient cross-presentation of incoming complement-opsonized virus could be initiated without infection of the DCs. CTLs generated by AT-2-HIV-C-exposed DCs elicited antiviral activity against HIV-infected target cells and the observed albeit weaker proliferation of the CD8^+^ T cells by repeated addition of AT-2-HIV-C-loaded DCs indicates a combination of infection and cross-presentation with respect to the effective CD8^+^ T cell response mediated by HIV-C-DCs.

To exclude that the CD8^+^ T cell induction is not due to triggering of DCs through CRs by complement itself, the specificity of the HIV-C-DC-primed CTLs was confirmed using peptide-tetramers and by HIV-specific lytic de-granulation and antiviral activity against autologous HIV-infected CD4^+^ T cells. The antigen-specificity of C-mediated CTL induction by DCs was further verified by using the FV model, since OVA-specific OT-1 T cells were not activated by F-MuLV- or F-MuLV-C-loaded bmDCs. Thus, compared to non-opsonized virus, C-opsonized virus activated DCs more efficiently to induce antigen-specific CTLs for both, HIV and FV. Our results indicate an improved processing of C-opsonized antigens due to putative binding to either CR3 (CD11b/CD18) or CR4 (CD11c/CD18), which are both expressed on DCs and may represent candidates for the attachment of C-opsonized pathogens. Very recently, Castro et al. [29] demonstrated that targeting Ags to CD11c, the □-chain of CR4, on DCs exerted uniquely effective properties to generate both CD4^+^ and CD8^+^ T cell responses, and suggested CD11c targeting as efficient vaccination approach for protection against tumor development. Furthermore, adenylate cyclase toxin (CyaA) of *Bordetella pertussis*, which is targeting CD11b/CD18 (CR3)-expressing cells, was successfully used to deliver CD8^+^ T cell epitopes for OVA as well as LCMV to induce specific CTL responses [30]–[32]. In contrast, CR3 ligation was also suggested to be associated with suppression of the stimulatory capacity of DCs and to provide a ‘non-danger’ signal to the cells, but in this particular study, CR3 was cross-linked via an anti-CR3 Ab rather than using a pathogen [33]. Skoberne et al. [34] suggested that under steady-state conditions, signaling via CRs makes DCs tolerogenic, which however, can be overcome by a significant inflammatory stimulus such as an infection. As observed in our experiments with DCs and naïve CD8^+^ T cells, C-opsonization of HIV/FV triggered an effective CTL response, which might be due to using a pathogen instead of an Ab to cross-link either CR3 or CR4 on the cell surface of DCs.

Although other antigen presenting cells expressing complement receptors like B cells and CD11b-positive monocytes/macrophages might be also involved in the generation of an antiviral CTL response in a complement dependent manner [35], [36], a study investigating the effect of DC-depletion on the induction of primary CTL responses in LCMV infection implied a crucial role of DCs in primary CTL response [26]. Despite we could not provide direct evidence, our *in vitro* and *in vivo* data suggest a link between complement and dendritic cells in the induction of retrovirus-specific CTLs.

To summarize, our *in vitro* and *in vivo* experiments indicate that HIV/FV potently activate DCs in a complement-dependent way to generate cellular immune responses. Aside from induction of retrovirus-specific CTLs by DCs in a complement-dependent manner, other CTL-generating mechanisms may act in concert, since the FV-specific CTL response does not completely disappear in C3-deficient mice. Nevertheless, our data emphasize an enhancing role of complement for the CTL- stimulatory capacity of DCs against retroviruses like HIV or FV. Understanding the exact interplay between differentially opsonized retroviral particles and APCs is of prime importance for DC-based vaccination strategies against retroviral infections.

## Materials and Methods

### Mice

Experiments were conducted using 3- to 6-month-old female C57BL/6 (B6) mice. B6 C3 knock-out (C3^−/−^) and FV-specific T cell receptor (TCR) transgenic (Tg) mice [37], carrying a TCR transgene that recognizes the *gag* leader peptide of FV [38], were also used. All mice were bred and maintained free of specific pathogens in the animal facility at the Department of Hygiene, Microbiology and Social Medicine, Section of Virology, Innsbruck, Austria. Mice were treated in accordance with the guidelines of the “European Convention for the Protection of Vertebrate Animals used for Experimental and other Scientific Purposes” and the Austrian law. Animal experiments were approved by the ethics committee of the Austrian Federal Ministry of Science and Research (BMWF-66.011/0081-II/10b/2008 and BMWF-66.011/0039-C/GT/2007).

### Virus propagation, purification and opsonization

Virus propagation and purification were performed as described [14]. Virus strains were obtained by the NIH-AIDS (available through WHO depositories) and following strains were used: BaL, 92UG037, 92BR030 (all R5), 93BR020 (R5/X4), NL4-3 (X4). Ultracentrifuged, concentrated virus was opsonized in 200µl with medium alone (HIV), heat-inactivated (thereby complement-inactivated) normal human serum (NHS) (HIV-hiC), or NHS as source for active complement (HIV-C). Subsequent to opsonization the different preparations were washed with 1 ml RPMI1640 w/o supplements (RPMI) and the virus was pelleted by ultracentrifugation. The virus was re-suspended in 200µl RPMI, aliquoted and the presence of C3 fragments or IgGs on the viral surface was confirmed by a virus capture assay (VCA) as shown in Supplementary Figure S1A. IgGs on the viral surface were also determined to exclude possible interactions of the virus preparations with FcRs expressed on DCs.

FV-B complex was obtained as 10% spleen homogenate of BALB/c mice 14 days post infection (dpi) and stored at −80°C. Virus preparations were thawed immediately before infection, centrifuged to pellet cell debris and diluted in phosphate-buffered saline (PBS). For all *in vivo* infection experiments, mice were injected intravenously with 10000 spleen focus-forming units (SFFU) of FV-complex in 0.3 ml PBS. Virus stocks were free of lactate dehydrogenase-elevating virus.

F-MuLV stocks for *in vitro* opsonization were generated in permissive *Mus dunni* cells. Virus containing cell-culture supernatants (SNs) were stored at −80°C until use. F-MuLV was opsonized in the presence of normal mouse serum (NMS) (as source of complement) at a dilution of 1∶10 for 60 minutes at 37°C (F-MuLV-C). As controls, F-MuLV incubated in medium alone or in heat-inactivated NMS (F-MuLV) was used. To remove NMS, virus was ultracentrifuged (23000×g, 2 hrs, 4°C) and the virus pellet was resuspended in medium. To confirm the opsonization pattern of the viruses, F-MuLV-C, and non-opsonized F-MuLV were applied in a VCA as previously described for HIV [39]. Real-time RT-PCR using FV-specific primers and fluorescently labelled Taq-Man probe was used to determine the amount of F-MuLV.

Since experiments using non-opsonized and heat-inactivated serum-opsonized virus preparations provided the same results, we decided to show only the results from non- opsonized HIV/F-MuLV.

### Generation of human monocyte-derived DCs and isolation of human CD4^+^ and CD8^+^ T cells

Monocytes were isolated from blood of normal healthy donors by using human CD14 MicroBeads (Miltenyi Biotec), according to the manufacturer's instructions. DCs were generated and analyzed as described [14]. B cells were depleted from PBLs using CD19 Pan B Dynabeads® (Dynal). Subsequently, CD4^+^ (>95% purity) and CD8^+^ (>94% purity) T cells were bead-purified and used with autologous DC's for the in vitro experiments. To avoid non-specific stimulation of the cells, all experiments were performed in the presence of human AB serum (IgG low, PromoCell) instead of FCS. Aliquots of DCs and T cells were frozen for boosting and rather functionality tests.

### Prime-boost experiments

Day 5 iDCs were stimulated with a cytokine-cocktail (IL-1β, IL-6, PGE_2_, TNF-α, IL-4, GM-CSF) for 24 hrs and then transferred into 96-well U-plates at a density of 10^4^/100 µl. DCs from all donors were loaded with 3 to 4 non- (HIV), complement-inactivated (HIV-hiC) or complement-opsonized (HIV-C) R5-, R5X4- and X4-tropic HIV strains for 3 hrs. DCs were exposed to 25 ng p24/ml of the differentially opsonized HIV (independent on using live or AT2-inactivated HIV-preparations). As a positive control for T cell proliferation, DCs were exposed to 1 µg/ml of the superantigen Staphylococcal Enterotoxin B (SEB, Sigma) for the same time-period. As additional controls iDCs and/or cytokine cocktail-stimulated mature DCs (mDCs) were included in the experimental settings. All tests were performed in triplicates. The cells were thoroughly washed and non-stimulated, naïve CD8^+^ T cells were added to the primed DCs at a ratio 3∶1. 25 U/ml IL-2 was added after 4 days and after another 4 days the T cells were again boosted with loaded DCs. This procedure was repeated 3 times and the CD8^+^ cells were analyzed for expansion, IFN-γ secretion, and functionality. HIV-induced proliferation of T cells was analyzed by FACS and IFN-γ secretion was detected using an IFN-γ OPTEia® kit according to the manufacturer's instructions (BD Biosciences) and in parallel an IFN-γ secretion assay (Miltenyi). The IFN-γ Secretion Assay enabled the sensitive detection and analysis of human IFN-γ-secreting cells by catching secreted IFN-γ on the CD8^+^ T cell surface by an immobilised IFN-γ-catch reagent. The CD8^+^ T cells were then boosted by loaded DCs and incubated at 37°C for 48 hrs. The secreted IFN-γ binds to the IFN-γ catch reagent on the cell surface of positive, secreting cells, which are subsequently labeled with an anti-IFN-γ antibody for sensitive detection by flow cytometry. The time point (48 hrs) for measurement of IFN-γ secretion turned out to be optimal for HIV-/HIV-C-DC-CD8^+^ T cells, but not for SEB-stimulated cells, which are recommended by the manufacturer to measure after an incubation period of 3–16 hrs. To perform tetramer analyses and cytotoxicity tests, T cells were expanded with the CD3/CD28 T cell expander (Dynal) according to the manufacturer's instructions following the third priming with loaded DCs.

### CD107a mobilization assay

Expanded CD8^+^ T cells were re-stimulated with CD3/CD28 beads and appropriate HIVgag peptides (P1: SLYNTVATL+CRQILGQLQPSLQTG [HLA-A02]), and control P2: WMTNNPPIPVGEIYK [HLA-A11/B035/Cw07]) in the presence of FITC-conjugated CD107a mAb [20] for 4 hrs at 37°C. The compatibility of epitopes and viruses used in these experiments (92BR030, 93BR020, NL4-3) was analyzed by the Epitope Location Finder (ELF) (http://www.hiv.lanl.gov/content/sequence/ELF/epitope_analyzer.html). In all experiments SEB (1 µg/ml; Sigma-Aldrich, St. Louis, MO) was used as a positive control for degranulation [21], and as negative control mDC-expanded CD8^+^ T cells were stimulated with the HIV-specific peptide P1 to control for spontaneous expression of CD107a [6]. FACS analyses were performed with additional markers (7-AAD, CD8-APC).

### Functionality of *in vitro* generated cytotoxic T cells

To further determine the functionality of the *in vitro* generated HIV-specific CTLs, frozen autologous CD4^+^ T cells were thawed, washed, and stimulated with CD3/CD28 beads (Dynal) in presence of IL-2 (100 U/ml). Pre-stimulation of the CD4^+^ T cells with CD3/CD28 was necessary to induce the up-regulation of CCR5. The *in vitro* primed and expanded CTLs were added to o/n HIV-incubated CD4^+^ T cells. In some experiments, primed CD8^+^ T cells were added after 3 days of infection of CD4^+^ T cells to see, if they can block an already ongoing infection. A reduction in virus production assessed by p24 ELISA on several days post addition of the expanded CTLs indicated an indirect confirmation of their functionality. By using this assay we were in addition able to circumvent the problem of the unknown HLA-type of the majority of the donors. This indirect confirmation of the functionality was performed with expanded CTLs from seven donors primed and boosted with HIV-exposed DCs.

### *In vitro* infection of bmDCs with differentially opsonized F-MuLV

BmDCs were generated as described by Inaba et al. [40] with some modifications. Briefly, 2×10^6^ bone marrow cells were cultivated in 10 ml of DC medium (RPMI 1640 supplemented with 10% FCS, 2 mM L-glutamine, 500 nM 2-ME, 100 U/mL penicillin/streptomycin and 1000 U/ml mouse GM-CSF) in Petri dishes for three days at 37°C. 10 ml of fresh DC medium was then added and cells were cultured for another 3 days. On day 6 non-adherent cells were removed, washed and cultivated in fresh 20 ml DC medium. Non-adherent cells obtained after 8 days displayed a myeloid DC phenotype (>85% CD11c and >95% CD11b) as revealed by flow cytometry.

### Co-culture of F-MuLV-loaded DCs with FV-specific TCRtg CD8^+^ T cells

For co-culture experiments, 1×10^6^ bmDCs were loaded with 1000 focus-forming units (FFU) F-MuLV. Since complement-opsonization might influence infectivity, bmDCs were loaded with equivalent amounts of F-MuLV-C RNA as determined by real-time RT-PCR. As controls, non-loaded bmDCs were used. After 24 hrs of incubation with differentially opsonized F-MuLV, bmDCs were washed twice and co-cultured with 1×10^6^ FV-specific CD8^+^ T cells isolated from the spleens of FV-specific TCRtg mice using the BD IMag CD8 T Lymphocyte Enrichment Set (BD Pharmingen) according to the manufacturer's instructions (purity >95% as determined by FACS). After 24 hrs of co-culture, activation of FV-specific CD8^+^ T cells was analyzed by flow cytometry. To study proliferation, isolated CD8^+^ T cells were labeled with CFSE prior to co-culture and the dilution pattern of CFSE was analyzed by FACS after 4 days.

To test whether DCs confronted with FV opsonized under *in vivo* conditions exert similar effects on FV-specific CD8^+^ T cells as bmDCs loaded with *in vitro* opsonized F-MuLV, B6 wild-type (wt) and B6 C3^−/−^ mice were infected i.v. with 10000 SFFU of the FV-complex. Single spleen cell suspensions from mice at 4 dpi were used to purify CD11c^+^ DCs using CD11c MicroBeads (Miltenyi) according to the manufacturer's instructions (purity ∼90% of CD11c^+^ cells). 1×10^6^ spleen DCs were then co-cultured for 24 hours with 1×10^6^ FV-specific TCRtg CD8^+^ T cells.

### FV infectious center (IC)-assays

Serial dilutions from the culture SNs of F-MuLV infected bmDCs were plated onto susceptible *Mus dunni* cells to detect productive infection of bmDCs *in vitro*. *Mus Dunni* cells were cultivated for 5 days, fixed with ethanol, stained with F-MuLV envelope-specific mAb 720 [41], and developed with goat anti-mouse peroxidase conjugate (DAKO) and with 3-amino-9-ethylcarbazole (AEC) substrate to detect *foci*.

### Tetramers and tetramer staining

To specifically characterise the generated CTLs, DCs and T cells from an HLA-A02^+^ donor were isolated. Following prime/boosting and expansion, cells (10^5^ cells/sample) were either stimulated with specific immunodominant HIV-1 gag epitopes P1 or P2 (see above). FACS analyses were performed with the HLA-A02-specific tetramer (SLYNTVATL-PE [Coulter]) and additional markers (7-AAD, CD8-APC).

To detect FV-virus-specific CD8^+^ T cells from FV-infected animals, 1×10^6^ spleen cells were stained with anti-mouse CD8 APC-labeled mAb (53-6.7, BD Pharmingen) and PE-labeled MHC class-I H-2D^b^ tetramers specific for the immunodominant GagL CTL epitope gPr80^gag^85–93 (Beckman Coulter), for 15 min at room temperature. The activated FV-specific CD8^+^ T cells were detected by co-staining with FITC-labeled anti-mouse CD43 mAb (1B11, BioLegend). Dead cells were excluded from analyses (BD Pharmingen).

### Cell surface staining

Expression of mouse cell-surface molecules was quantified using following BD Pharmingen monoclonal antibodies (mAbs): anti-CD8-APC, anti-CD69-PE, anti-CD25-FITC, CD11b-PE, CD11c-APC. To detect FV-infected cells, spleen cells from FV-infected mice were stained with biotinylated mAb clone#34. Binding of biotinylated mAb 34 was detected with APC-conjugated streptavidine (BD Pharmingen). For flow cytometry, 1×10^6^ cells were incubated with 1 to 5µg/ml mAb for 30 min at 4°C, washed twice in PBS/1% FCS, and measured on a FACSCanto II (BD). Dead cells were again excluded using 7-AAD. Data were analyzed using FACS Diva (BD).

### Statistical analysis

Differences between samples were analyzed by the GraphPad prism software. P values p<0.05 in the unpaired Student's t-test were scored as significant.

## Supporting Information

Figure S1 *Figure S1A. Virus capture assay (VCA) of differentially opsonized HIV-preparations.* HIV was opsonized with medium alone (HIV), normal human serum (NHS) as source of complement (HIV-C), or complement-inactivated NHS (HIV-hiC). Following opsonization the virus preparations were washed, centrifuged at 14000 rpm/90min/4°C, and the pellet was resuspended in 200µl RPMI. The opsonization pattern was determined by VCA using an anti-human C3c/C3d-, IgG or an anti-mouse IgG as control. As expected no C3-fragments or IgGs were found on the HIV and HIV-hiC virus preparations. C3 fragments, but no IgGs were detected on HIV-C. *Figure S1B. DCs loaded with complement-inactivated NHS do not induce proliferation of CD8^+^ T cells.* Percentages of CFSE- [proliferated]/CD8^+^ T cells of SEB-, HIV-, HIV-C-, or HIV-hiC-DC-primed CD8^+^ T cells from one representative virus preparation are outlined in this Figure. Only SEB- and HIV-C-DCs induced high expansion of CD8^+^ T cells, while only low T cell proliferation was observed upon stimulation with HIV-, or HIV-hiC-DCs. Only low T cell expansion was also detected when priming the cells with DCs loaded with IgG-opsonized HIV (not shown). All experiments were performed with DCs exposed to HIV and HIV-hiC. Since those preparations exerted similar effects in all experiments and to simplify the terminology and figures, we only showed HIV in all Figures.
(0.14 MB PPT)

Figure S2 *AT2-HIV-C-DCs exert an antiviral effect upon co-culture with infected, autologous CD4^+^ T cells.* Expanded AT2-HIV-C-DC-primed CD8^+^ T cells proved to be functional upon addition to infected, autologous CD4^+^ T cells and significantly inhibited productive infection compared to CD8^+^ T cells primed/boosted with AT2-HIV-DCs (p = 0.02). Too, infection of CD4^+^ T cells was higher in co-cultures, where SEB-, mDC- or iDC-primed CD8^+^ T cells were added. This experiment was performed in triplicates with 3 different AT2-inactivated HIV strains (BaL, 92UG037, 93BR020) and mean values are shown.
(0.12 MB PPT)

Figure S3 *Expanded HIV-C-DC-CD8^+^ T cells exert an antiviral effect on autologous CD4^+^ TCs infected with HIV 3 days prior addition.* An already on-going infection of CD4^+^ TCs (3 days pre-infected) was inhibited by addition of HIV-C-DC-primed CD8^+^ T cells as shown by determining p24 values from the supernatants 5 and 9 days post addition of CD8^+^ T cells. In contrast, HIV-DC-primed CD8^+^ T cells did not show an antiviral effect. This experiment was performed in triplicates with cells from 2 donors and mean values are given.
(0.11 MB PPT)
