# Supplementary figures and images for: Complement as an Endogenous Adjuvant for Dendritic Cell-Mediated Induction of Retrovirus-Specific CTLs
Source: PLoS Pathog. 2010 Apr 29;6(4):e1000891. doi: 10.1371/journal.ppat.1000891 (PMC2861708; doi:10.1371/journal.ppat.1000891)

## Slide 1
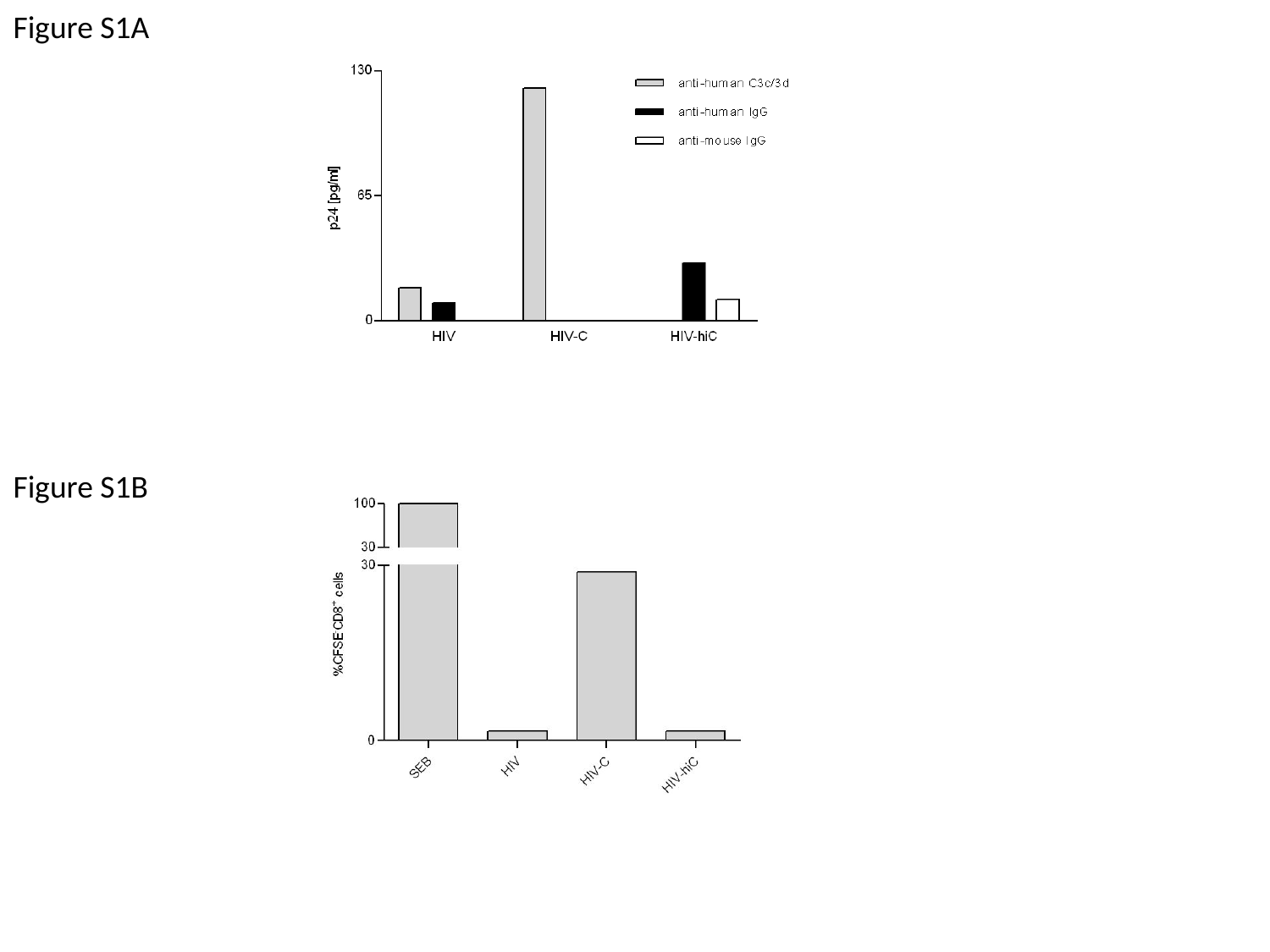

Figure S1A
Figure S1B

Supplement: Figure S1 — A. Virus capture assay (VCA) of differentially opsonized HIV-preparations. HIV was opsonized with medium alone (HIV), normal human serum (NHS) as source of complement (HIV-C), or complement-inactivated NHS (HIV-hiC). Following opsonization the virus preparations were washed, centrifuged at 14000 rpm/90min/4°C, and the pellet was resuspended in 200µl RPMI. The opsonization pattern was determined by VCA using an anti-human C3c/C3d-, IgG or an anti-mouse IgG as control. As expected no C3-fragments or IgGs were found on the HIV and HIV-hiC virus preparations. C3 fragments, but no IgGs were detected on HIV-C. Figure S1B. DCs loaded with complement-inactivated NHS do not induce proliferation of CD8+ T cells. Percentages of CFSE- [proliferated]/CD8+ T cells of SEB-, HIV-, HIV-C-, or HIV-hiC-DC-primed CD8+ T cells from one representative virus preparation are outlined in this Figure. Only SEB- and HIV-C-DCs induced high expansion of CD8+ T cells, while only low T cell proliferation was observed upon stimulation with HIV-, or HIV-hiC-DCs. Only low T cell expansion was also detected when priming the cells with DCs loaded with IgG-opsonized HIV (not shown). All experiments were performed with DCs exposed to HIV and HIV-hiC. Since those preparations exerted similar effects in all experiments and to simplify the terminology and figures, we only showed HIV in all Figures. (0.14 MB PPT) [file ppat.1000891.s001.ppt]

## Slide 1
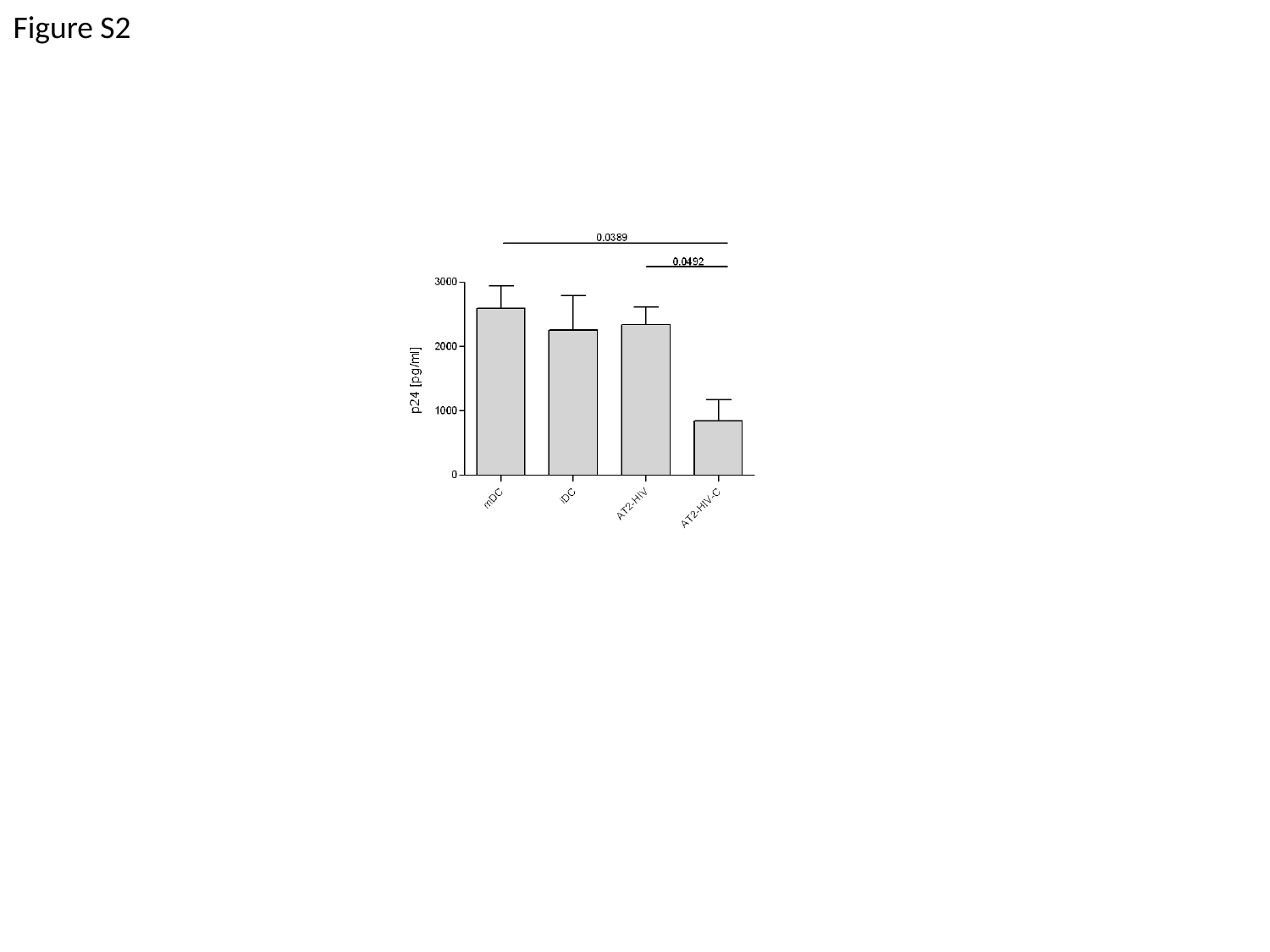

Figure S2

Supplement: Figure S2 — AT2-HIV-C-DCs exert an antiviral effect upon co-culture with infected, autologous CD4+ T cells. Expanded AT2-HIV-C-DC-primed CD8+ T cells proved to be functional upon addition to infected, autologous CD4+ T cells and significantly inhibited productive infection compared to CD8+ T cells primed/boosted with AT2-HIV-DCs (p = 0.02). Too, infection of CD4+ T cells was higher in co-cultures, where SEB-, mDC- or iDC-primed CD8+ T cells were added. This experiment was performed in triplicates with 3 different AT2-inactivated HIV strains (BaL, 92UG037, 93BR020) and mean values are shown. (0.12 MB PPT) [file ppat.1000891.s002.ppt]

## Slide 1
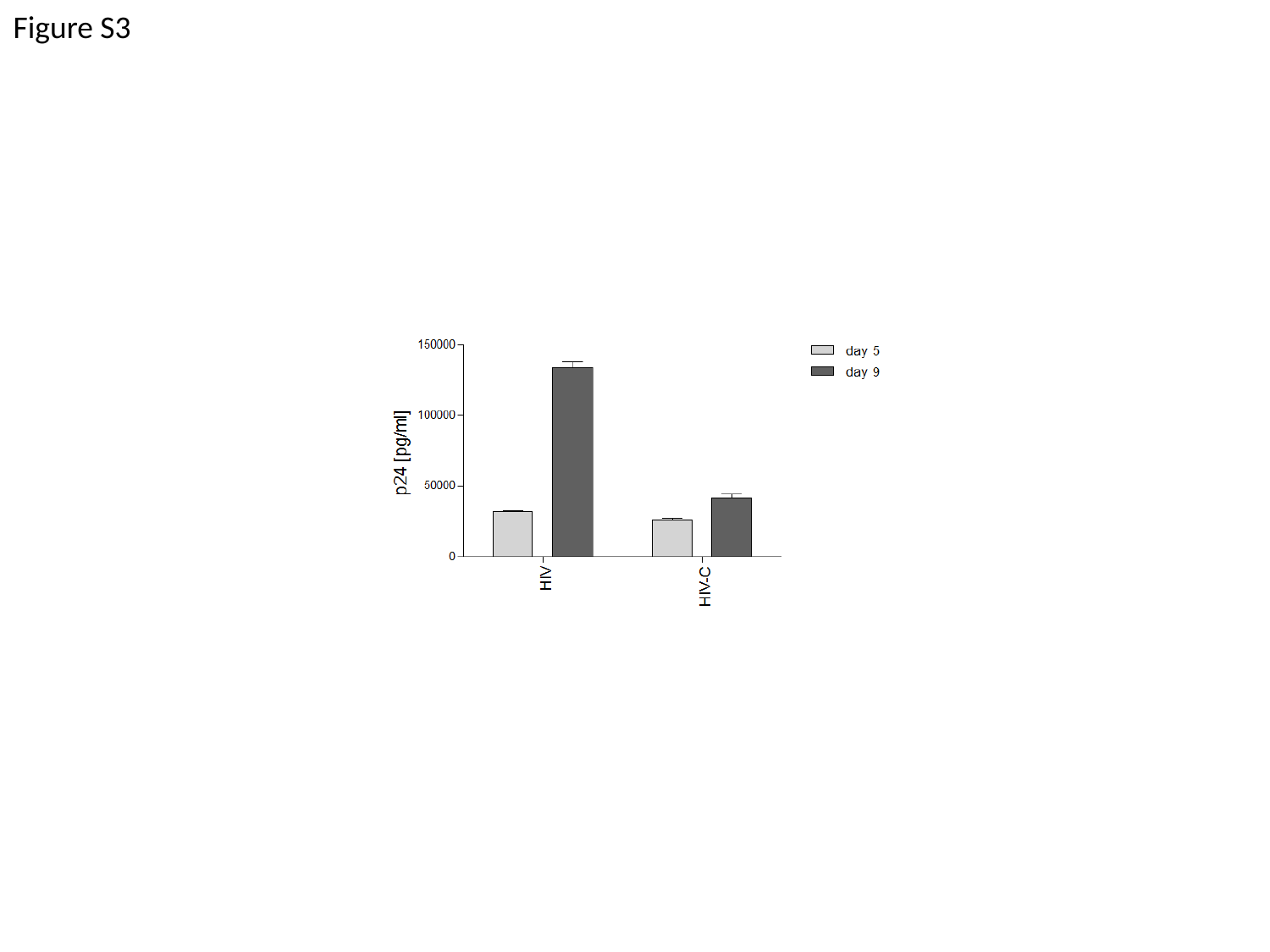

Figure S3

Supplement: Figure S3 — Expanded HIV-C-DC-CD8+ T cells exert an antiviral effect on autologous CD4+ TCs infected with HIV 3 days prior addition. An already on-going infection of CD4+ TCs (3 days pre-infected) was inhibited by addition of HIV-C-DC-primed CD8+ T cells as shown by determining p24 values from the supernatants 5 and 9 days post addition of CD8+ T cells. In contrast, HIV-DC-primed CD8+ T cells did not show an antiviral effect. This experiment was performed in triplicates with cells from 2 donors and mean values are given. (0.11 MB PPT) [file ppat.1000891.s003.ppt]
